# Abdominal wound length influences the postoperative serum level of interleukin-6 and recovery of flatus passage among patients with colorectal cancer

**DOI:** 10.3389/fsurg.2024.1400264

**Published:** 2024-06-24

**Authors:** Po-Li Tsai, Jian-Syun Chen, Chi-Hsin Lin, Tzu-Chi Hsu, Yu-Wen Lin, Ming-Jen Chen

**Affiliations:** ^1^ Department of Surgery, Division of Colorectal Surgery, Mackay Memorial Hospital, New Taipei City, Taiwan; ^2^ Department of Medical Research, Mackay Memorial Hospital, New Taipei City, Taiwan; ^3^ Department of Bioscience Technology, Chung Yuan Christian University, Taoyuan, Taiwan; ^4^ Department of Medicine and Institute of Biomedical Sciences, Mackay Medical College, New Taipei City, Taiwan

**Keywords:** abdominal wound length, interleukin 6 (IL-6), first flatus passage, hospital stay, colorectal cancer (CRC)

## Abstract

**Introduction:**

A mini-laparotomy for colorectal cancer (CRC) has been reported to shorten postoperative ileus (POI) and hospital stay. Interleukin-6 (IL-6) plays a role in intestinal tissue inflammation, leading to POI. This study investigated the effects of abdominal wounds and IL-6 levels on POI in patients having CRC surgery.

**Materials and methods:**

Forty-three patients with CRC underwent bowel resection. Serum samples were collected preoperatively and at 2, 24, and 48 h after surgery for cytokine quantification by ELISA. Clinical data, including time from surgery to first passage of flatus and postoperative hospital stay, demographic and pathological data, and routine blood tests, were compared statistically with abdominal wound length and the postoperative increments of cytokines (designated as Δ).

**Results:**

The length of the abdominal wound showed a significant correlation with clinical variables (length of operation time, time of first flatus passage, and length of postoperative hospital stay) and cytokine variables (IL-6(Δ2 h), IL-8(Δ2 h) and IL-10(Δ2 h). Linear regression analysis showed that the abdominal wound length significantly influenced the operation time, time of first flatus passage, and length of postoperative hospital stay (*p* < 0.001). The length of the abdominal wound showed a significant influence on the IL-6(Δ2 h) and IL-8(Δ2 h) (*p* < 0.001, respectively) but no influence on IL-10(Δ2 h). IL-6(Δ2 h), but not IL-8(Δ2 h), significantly influenced the time to first flatus passage and length of hospital stay (*p* = 0.007, *p *= 0.006, respectively). The mini-laparotomy approach (wound length <7 cm) led to significantly shortened operation time, time of first flatus passage, length of postoperative stay (*p* = 0.004, *p* = 0.003, *p* = 0.006, respectively) as well as reduced postoperative increment of IL-6(Δ2 h) (*p* = 0.015). The mini-laparotomy for anterior resection surgery significantly influenced operation time, time of first passage of flatus, length of postoperative stay, and IL-6(Δ2 h).

**Conclusion:**

Our study is the first to report the complex interaction among the length of the abdominal wound, IL-6 serum level, recovery of the first passage of flatus, and postoperative hospital stay. These results suggest that smaller abdominal wounds and smaller postoperative IL-6 increments were associated with faster recovery of flatus passage and shorter hospital stays.

## 1 Introduction

Postoperative ileus (POI), which occurs after major abdominal operations, such as colorectal cancer resection, is considered a physiological cessation of bowel motility during transit. The estimated duration of this temporary POI is between 24 and 48 h in the stomach, less than 24 h in the small intestine, and 48–72 h in the colon (1). However, the recovery of colonic motility can be delayed by surgical procedures involving colon resection with anastomosis compared to other types of abdominal surgery without colonic anastomosis (2). The resolution of POI has been assessed using bowel sounds, flatus passage, defecation, tolerance to meals, and myoelectric activities of smooth muscle as indicators of bowel motility recovery in different studies (3, 4). Thus, the criteria to define prolonged POI in clinical studies included delayed recovery of bowel motility (e.g., passage of flatus or defecation) and/or associated abdominal symptoms (e.g., intolerance to diet, abdominal distension, and nausea/vomiting). The duration of such criteria was defined as three to seven days in different studies (5). Clinically important, prolonged POI is associated with longer hospital stays and increased medical costs after colonic surgery (6–8).

The evidence indicates that POI mechanisms are multifactorial. Animal experiments and human studies have shown that both local inflammation of the bowel wall and inflammatory cytokines in the systemic circulation may play active roles in developing POI. Interleukin-6 (IL-6) is a proinflammatory cytokine and is among the most studied cytokines. In animal experiments, direct trauma to the bowel wall has been shown to cause a local inflammatory response in the muscularis propria, including inflammatory cell infiltration and elevated cellular expression of proinflammatory cytokines, including IL-6 (9, 10). Similar phenomena were observed in a study of human bowel tissues (11). In an animal experiment, abdominal laparotomy induced elevated serum IL-6 levels and inhibition of gastrointestinal transit in the same time course (12). In a human study, elevated serum IL-6 levels after abdominal surgery were involved in delayed recovery of gastric emptying and impaired gastric electrical activity in patients with postoperative ileus (13, 14). Although these data have indicated the possible role of IL-6 in the development of POI, there is still a lack of clinical studies focusing on the association between postoperative alterations in IL-6 and the recovery of colonic motility of transit among patients undergoing surgical resection for colorectal cancer.

To date, only a few studies have reported that IL-8 and IL-10 may have biological functions related to bowel motility. In an animal study, proinflammatory IL-8 enhanced the contractile response of the small intestine to stimulation by acetylcholine (15). IL-10 is an anti-inflammatory cytokine. In an animal model of inflammatory bowel disease, the colonic smooth muscle of IL-10 knockout mice showed decreased contractility relative to the colonic smooth muscle of wild-type IL-10 mice (16). In contrast, the elevation of serum IL-10 levels elicited by peritoneal air exposure was correlated with a decrease in gastrointestinal transit in an animal study (12). These reports suggest a potential influence of IL-8 and IL-10 on bowel motility; however, the observed functions of IL-10 are contradictory.

In recent years, an increasing number of studies have reported the clinical benefits of mini-laparotomies for colorectal cancer resection. Different authors defined a mini-laparotomy as an abdominal wound length either less than 7 or 8 cm (17–20). Regardless of the mild difference in wound length, the mini-laparotomy was significantly associated with earlier recovery of flatus passage than a conventional laparotomy (17, 20). A recent case-controlled study comparing the medical costs of a mini-laparotomy (less than 8 cm) and a conventional laparotomy in patients with colorectal cancer reported that the mini-laparotomy is more cost-effective because of fewer complications and a shorter post-operative hospital stay (19). Thus far, the biological mechanism underlying the benefits of mini-laparotomy abdominal wounds on the earlier recovery of flatus passage or shorter hospital stay remains to be investigated.

Based on the current data, we hypothesized that the length of the abdominal wound influences the postoperative systemic cytokine response (IL-6) and clinical outcomes (including recovery of colonic motility and postoperative hospital stay). The postoperative IL-6 response may also be involved in the complex interaction between abdominal wounds and clinical outcomes.

In the present study, we clarified the complex interactions between abdominal wound length, postoperative IL-6 levels, and recovery of colon motility. We demonstrated the beneficial effect of the mini-laparotomy approach in decreasing the postoperative IL-6 cytokine response and shortening the duration of recovery from flatus passage and postoperative hospital stay. In addition, we demonstrated that postoperative IL-8 and IL-10 did not influence the recovery of colon motility.

## 2 Materials and methods

### 2.1 Patients

This study retrospectively analyzed prospectively collected data from 45 patients. After obtaining approval from the Institutional Review Board (MMH-I-S-154), the patients were enrolled in the study between September 2005 and February 2007. Patients with complicated conditions, including perforation, peritonitis, preexisting infection, poor nutrition, and severe comorbidities, were excluded. All enrolled patients signed an informed consent form to check their serum cytokine levels before and after surgery. Of the 45 patients, two were not included in the data analysis after entering the study because of anastomotic leakage and unexpected colostomy. The remaining 43 patients who had resectable colorectal cancer received open abdominal approach to remove the primary tumor. Among them, five patients with synchronous metastasis to liver (two cases), lung (one case), omentum (one case) and small bowel mesentery (one patient) only had resection of the primary tumor. We did not include patients who received laparoscopic approach or robotic approach for tumor resection to compare with patients having open approach. The patients underwent surgery at the Department of Surgery, Division of Colorectal Surgery, Mackay Memorial Hospital, by two surgeons who reached a consensus on the study design, surgical procedures, and postoperative care. The abdominal wound length was determined based on the surgeon's judgment according to clinical indications, including tumor size and an adequate operation field to dissect the mesentery and feeding vessels. In this study, an abdominal wound length of <7 cm was defined as a mini-laparotomy (17, 18).

The demographic and clinical variables were prospectively collected, including age, gender, body mass index (BMI), abdominal wound length, operation time, blood loss, tumor characteristics, tumor locations, anesthetic methods, postoperative analgesic methods, the first recovery of flatus passage, the postoperative hospital stay, the routine preoperative data from blood tests.

### 2.2 Measurement of serum cytokine levels

The timing of blood collection and quantification of cytokine levels in blood samples have been described previously (21). Serum samples were collected before surgery and at indicated time points. Serum levels of IL-6, IL-8, and IL-10 were measured using an ELISA kit, according to the manufacturer's instruction (R&D Systems, Minneapolis, MN, USA).

All serum samples were measured within the range of the standard curve of the ELISA kit: 0–300 pg/ml for IL-6 (kit D6050, R&D Systems); 0–2,000 pg/ml for IL 8 (kit D8000C, R&D Systems); and 0–500 pg/ml for IL-10 (kit D1000B, R&D Systems). The cytokine concentration was considered zero when the sample concentration was below the measurable limit.

To minimize the effect of individual physiological variations between patients in this study, the postoperative change (designated Δ) in cytokines was calculated by the following formula: cytokine (Δ time point) = postoperative value of a cytokine at an indicated time–the paired preoperative cytokine value from the same patient. Therefore, the individual variations among patients were normalized, and the data for cytokine preoperative baseline level, Δ2 h, Δ24 h, and Δ48 h were used for comparative analysis.

### 2.3 Statistics

Correlations between variables were analyzed using Spearman's correlation analysis. The causation between variables was analyzed using linear regression analysis. Differences in variables between the two groups were analyzed using the Mann-Whitney *U*-test. The Kruskal Wallis test was used for comparison between multiple groups. Categorical variables were compared using the chi-squared test (Fisher's exact test). Values of *p* < 0.05 were considered statistically significant.

## 3 Results

### 3.1 Surgical stress of colorectal cancer (CRC) resection elicited elevation of serum cytokines

Table 1 shows the clinical variables and characteristics of the enrolled patients. There were 21 females and 22 males with a mean age of 60.3 ± 10.7 years old. Eighteen patients (42%) had colon cancer and 25 (58%) had rectal cancer. The mean length of the abdominal incision wound was 10.2 ± 4.8 cm. The mean operation time was 186.8 ± 88.3 min. The mean blood loss was 204.7 ± 158.8 ml. The mean BMI was 23.5 ± 3.1 (kg/m^2^). The mean duration from operation to first flatus passage was 3.2 ± 1.3 days, and the mean duration of postoperative hospital stay was 10.9 ± 4.5 days. All patients recovered from the surgery smoothly without any remarkable complications.

**Table 1:** Clinical variables of enrolled patients.

Clinical variables	Number (*N* = 43)
Age (years)	
Mean ± SD	60.3 ± 10.7
Median(IQR)	59.0 (11.0)
Gender (*n*, %)	
Male	22 (51.2%)
Female	21 (48.8%)
Site of cancer	
Appendix	1 (2.3%)
Cecum	1 (2.3%)
Ascending	1 (2.3%)
Cecum + ascending	1 (2.3%)^a^
Transverse	2 (4.7%)
Descending	1 (2.3%)
Sigmoid	11 (23.3%)
Rectum	25 (60.4%)
Dukes’ stage (*n*, %)	
A	3 (7%)
B	10 (23.3%)
C	26 (60.5%)
D	4 (9.3%)
Body mass index (kg/m^2^)	
Mean ± SD	23.5 ± 3.1
Median(IQR)	23.4 (5.0)
Wound length (cm)	
Mean ± SD	10.2 ± 4.8
Median(IQR)	7.0 (7.7)
Operation time (min)	
Mean ± SD	186.8 ± 88.3
Median(IQR)	165.0 (75.0)
Blood loss (ml)	
Mean ± SD	204.7 ± 158.8
Median(IQR)	200.0 (200.0)
Anesthesia	
General(intubation)	43 cases
Postoperative analgesia	
PCA	36
Non-PCA	7
First passage of flatus (day)	
Mean ± SD	3.2 ± 1.3
Median(IQR)	3.0 (2.0)
Postoperative hospital stay (day)	
Mean ± SD	10.9 ± 4.5
Median(IQR)	9.0 (4.0)

Continuous data are expressed as mean ± SD and median(IQR); y/o, years old; cm, centimeter; min, minutes; ml, milli-liter; PCA, Patient-controlled analgesia with morphine; Non-PCA, Demerol injection.

^a^ One patient had synchronous at cecal and A-colon cancers. First flatus passage: time from operation to first flatus; Length of postoperative stay: time from operation to discharge.

**Figure 1:**
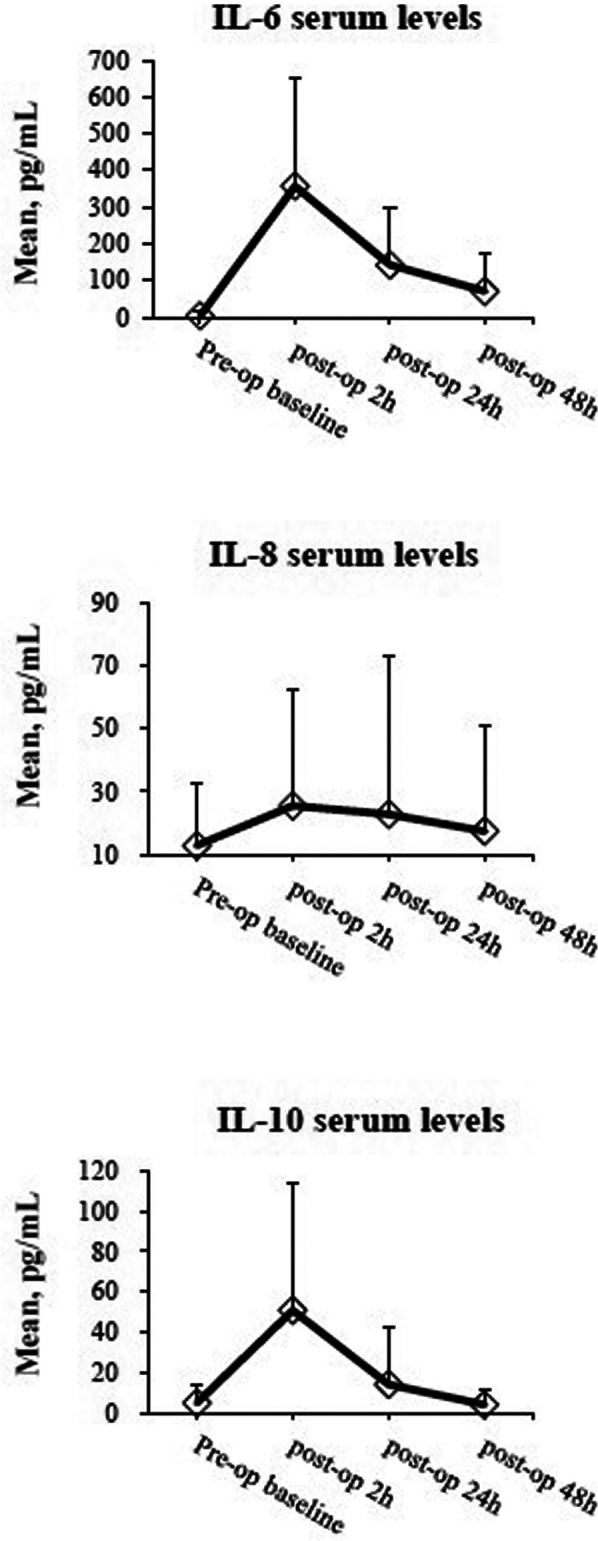
The mean serum levels of IL-6, IL-8, and IL-10 are shown in time course. Briefly, cytokines were quantified by ELISA assay on serum taken preoperatively at 2, 24, and 48 h after surgery. Data are expressed as mean ± standard deviation.

Figure 1 shows the mean levels of IL-6, IL-8, and IL-10 2, 24, and 48 h after colorectal cancer surgery. The highest postoperative levels of the three cytokines occurred at 2 h after operation (Δ2 h). The median serum cytokine levels are shown in Supplementary Figure S1.

### 3.2 Interaction between abdominal wound length, postoperative increments of cytokines, and recovery of flatus passage

Table 2 shows that abdominal wound length was positively correlated with clinical outcomes, including operation time (*p* < 0.001), time to first passage of flatus (*p* < 0.001), and duration of postoperative hospital stay (*p* < 0.001). The length of the abdominal wound was also positively correlated with the postoperative increments of IL-6 (Δ2 h), IL-8 (Δ2 h) and IL-10(Δ2 h) (*p* = 0.001, *p* = 0.016, *p* = 0.003, respectively).

**Table 2:** Correlation analysis between abdominal wound length and variables.

Variables	Spearman's coefficients (*ρ*)	*p* values
Age (y/o)	0.183	0.240
BMI	−0.219	0.158
Operation time (min)	0.594	**<0.001**
Blood loss (ml)	−0.017	0.913
First passage of flatus (day)	0.519	**<0.001**
Postoperative hospital stay (day)	0.610	**<0.001**
IL-6 (pg/ml)		
pre-op	0.151	0.335
Δ2 h	0.480	**0**.**001**
Δ24 h	0.077	0.625
Δ48h	0.227	0.142
IL-8 (pg/ml)		
pre-op	0.102	0.516
Δ2 h	0.371	**0**.**016**
Δ24 h	0.057	0.716
Δ48 h	0.203	0.193
IL-10 (pg/ml)		
pre-op	0.076	0.628
Δ2 h	0.443	**0**.**003**
Δ24 h	0.148	0.345
Δ48 h	−0.121	0.441

First flatus passage: time from operation to first flatus. Length of postoperative stay: time from operation to discharge.

Significant differences in statistical comparisons are shown by bold values.

Linear regression analysis was performed to verify whether the abdominal wound length had a causative effect on the correlated variables (Table 3). The results showed that the length of the abdominal wound significantly influenced the operation time, recovery of the first passage of flatus, and length of postoperative hospital stay (*p* < 0.001, respectively). The length of the abdominal wound also showed a significant influence on the increment magnitude of IL-6 (Δ2 h) and IL-8 (Δ2 h) (*p* < 0.001, respectively) but no influence on the increment of IL-10(Δ2 h) (*p* = 0.069).

**Table 3:** Linear regression of wound length on cytokines and clinical outcomes.

Variables	R	Unadjusted coefficients β values	*p* values
Operation time (min)	0.710	13.148	**<0.001**
First flatus passage (day)	0.574	0.151	**<0.001**
Length of postoperative stay (day)	0.672	0.628	**<0.001**
IL-6 (Δ2 h)	0.582	35.757	**<0.001**
IL-8 (Δ2 h)	0.581	3.412	**<0.001**
IL-10 (Δ2 h)	0.284	3.522	0.069

Significant differences in statistical comparisons are shown by bold values.

Linear regression analysis was further performed to examine whether the magnitudes of IL-6 (Δ2 h) and IL-8 (Δ2 h) have a causative influence on the recovery of first flatus passage and postoperative hospital stay. Table 4 shows that the increment magnitude of IL-6 (Δ2 h) significantly influenced the recovery of flatus passage and length of hospital stay (*p* = 0.007 & *p *= 0.006, respectively). However, IL-8 (Δ2 h) did not significantly influence these clinical outcomes.

**Table 4:** Linear regression of cytokine increments on clinical outcomes.

Variables	R	Unstandardized coefficients β values	*p* values
Outcome 1: recovery of flatus passage			** **
IL-6(Δ2 h)	0.413	0.002	**0**.**007**
IL-8(Δ2 h)	0.203	0.010	0.197
Outcome 2: postoperative hospital stay			** **
IL-6(Δ2 h)	0.414	0.006	**0**.**006**
IL-8(Δ2 h)	0.243	0.039	0.121

Significant differences in statistical comparisons are shown by bold values.

Furthermore, linear regression analysis showed that the recovery of first flatus passage significantly influences the postoperative hospital stay (R = 1.000, unadjusted coefficient β = 1.012, *p* < 0.001).

**Figure 2:**
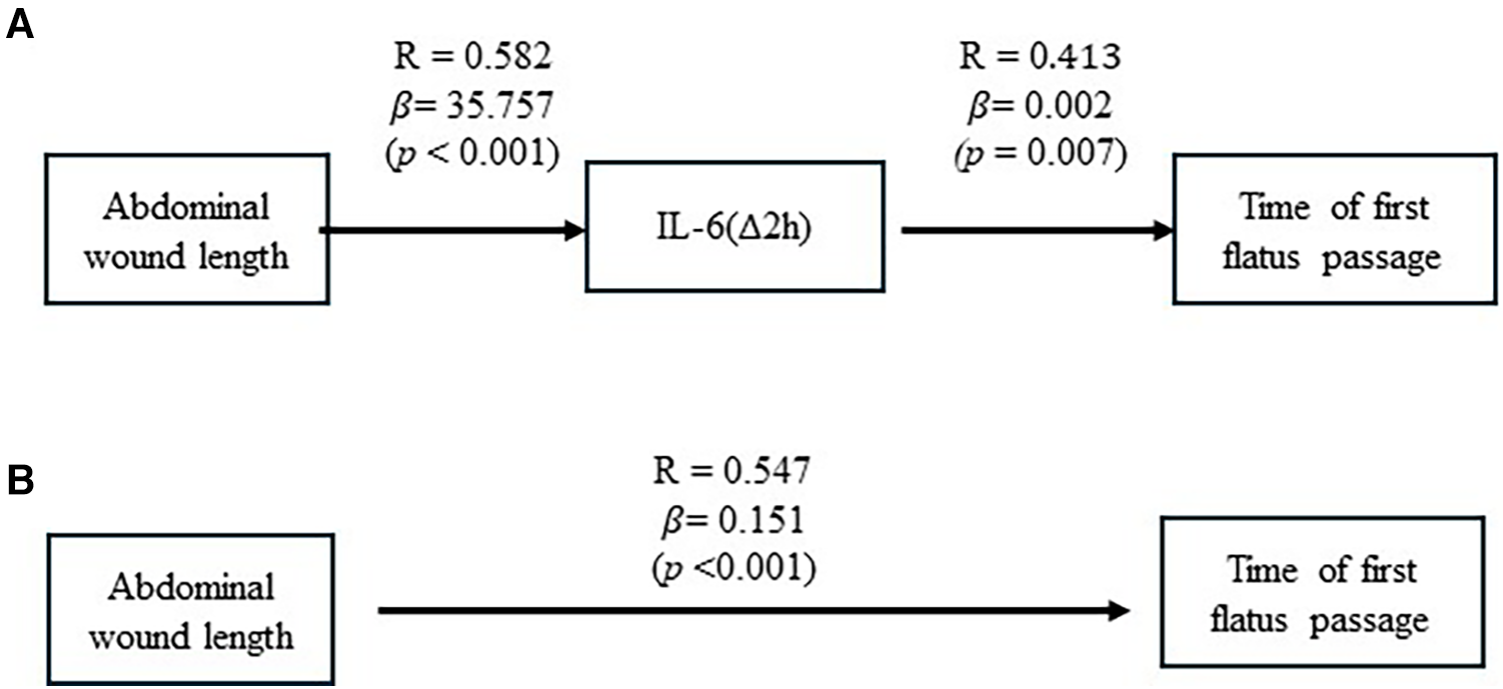
The causative effect of abdominal wound length on posterior IL-6(Δ2 h) increment and recovery of flatus passage. (**A**) Results of linear regression analysis showed the causative effect of abdominal wound length on postoperative IL-6(Δ2 h) increment and the causative effect of postoperative IL-6(Δ2 h) increment on recovery of flatus passage. (**B**) Results of linear regression analysis were illustrated to show the causative effect of abdominal wound length on recovery of flatus passage.

By taking these analyzed results together, we summarized an interaction between abdominal wound length, postoperative increments of IL-6 (Δ2 h), and recovery of first flatus passage in Figure 2. Figure 2A estimated that a one-centimeter length of abdominal wound was associated with a 35.8 pg/ml increment of IL-6 (Δ2 h). In turn, a 1 pg/ml increment of IL-6 (Δ2 h) was associated with 0.002 days of waiting time for the first passage of flatus. Meanwhile, the abdominal wound could influence, through other pathways, the recovery of the first flatus passage, with one centimeter of wound length associated with 0.151 days of waiting time to have the first passage of flatus (Figure 2B). A waiting time of one day for the first flatus passage was associated with 1.012 days postoperative hospital stay.

### 3.3 Effects of mini-laparotomy on clinical outcomes and IL-6 increments

Having verified the effects of abdominal wound length and IL-6 levels on clinical outcomes, we further examined the clinical benefits of mini-laparotomy (wound size <7 cm) in our patients. In Table 5, the median abdominal wound length in the mini-laparotomy group was 6.8 cm (range: 5.8–7, IQR: 0.3), compared to a median abdominal wound of 14.5 cm (range: 7.5–24, IQR:7.0) in the non-mini-laparotomy group. The operation time, time to first passage of flatus, and length of postoperative stay were shorter in the mini-laparotomy group (*p* = 0.004, *p* = 0.003, *p* = 0.006, respectively). The postoperative increments of IL-6(Δ2 h) in the mini-laparotomy group were significantly less than the increment in the non-mini-laparotomy group (*p* = 0.015).

**Table 5:** Effects of mini-laparotomy on patients receiving resection of colorectal cancer.

Variables	Wound length <7 cm	Wound length >7 cm	*p* values
Age (years)			
Median	56 (13)	62 (8)	0.394
Range	47–85	32–84	
Gender (M/F)	11/11	10/11	1.000
Cancer stage			0.914
Dukes’ A	1	2	
Dukes’ B	6	4	
Dukes’ C	13	13	
Dukes’ D	2	2	
Site of cancer			0.288
BMI	23.52 (5.00)	23.40 (4.00)	0.194
Wound length (cm)	6.8 (0.3)	14.5 (7.0)	**<0.001**
Operation time (min)	150 (50)	205 (91)	**0**.**004**
Blood loss (ml)	200 (213)	200 (175)	0.950
Postoperative analgesia			0.412
PCA	17	19	
Non-PCA	5	2	
First passage of flatus	2 (2)	4 (2)	**0**.**003**
Postoperative hospital stay	9 (2)	12 (10)	**0**.**006**
IL-6 (pg/ml)			
pre-op	0 (3.02)	0 (3.74)	0.669
Δ2 h	207.51 (205.19)	396.13 (488.65)	**0**.**015**
Δ24 h	103.53 (184.07)	63.13 (181.69)	0.451
Δ48 h	37.45 (49.34)	43.69(65.72)	0.536

Continuous data are expressed as median(IQR); y/o, years old; cm, centimeter; min, minutes; ml, milliliter; BMI, body mass index; PCA, Patient-controlled analgesia; Non-PCA, demerol injection; Site of cancer, sites similar to those in Table 1. Mann-Whitney *U*-test for continuous data; Chi-square test for categorical data.

Significant differences in statistical comparisons are shown by bold values.

Clinical variables, including age, sex, cancer stage, cancer sites, amount of blood loss, BMI, and methods of postoperative analgesia, showed no statistical differences between the two groups (Table 5). The preoperative blood test results of the two groups did not show significant differences (Table 6).

**Table 6:** Clinical variables of patients receiving mini-laparotomy for colorectal cancer.

Variables	Wound length <7 cm	Wound length >7 cm	*p* values
Hemoglobin	13.15 (3.15)	12.60 (4.45)	0.481
WBC	6,600 (2,225)	6,650 (2,275)	0.601
Neutro percent	63.65 (16.98)	61.45 (19.23)	0.285
Neutrocyte count	4,226 (1,673.72)	4,351 (2,460.78)	0.903
Lympho percent	27.10 (14.3)	29.30 (18.53)	0.198
Lymphocyte count	1,615.5 (1,035.75)	1,749.7 (1,148.38)	0.285
Platelet count	307,000 (112,250)	353,500 (226,000)	0.302
Albumin	3.85 (0.5)	3.85 (0.48)	0.614
Total bilirubin	0.20 (0.4)	0.40 (0.48)	0.107
Alk-p-tase	74.50 (36.25)	71.00 (19.75)	0.734
GOT	21.50 (7.75)	25.50 (13.00)	0.199
GPT	19.00 (9.75)	20.00 (15.5)	0.764
Creatinine	0.90 (0.20)	0.90 (0.18)	0.334
BUN	10.50 (3.75)	9.50 (8.00)	0.826
CEA	2.23 (9.76)	2.02 (1.97)	0.957
CRP			
Pre-op	0.275 (0.60)	0.38 (0.35)	0.667
Δ48 h	8.82 (8.39)	9.23 (9.99)	0.639
Δ7 day	0.395 (1.58)	0.29 (0.83)	0.523

Data of continuous variables were expressed as median (IQR). Mann-Whitney *U*-test for continuous data.

### 3.4 Effects of mini-laparotomy among patients receiving anterior resection

One may wonder that the site of mesenteric dissection or surgery involving the small intestine may also influence the IL-6 response and clinical outcomes. Thus, we analyzed the data of patients who had surgical dissection limited to the mesentery of the inferior mesenteric artery and no surgery on the small bowel and investigated the effects of mini-laparotomy on patients who underwent anterior resection for cancer of the sigmoid and rectum. Table 7 shows that the operation time, time to first passage of flatus, and length of postoperative stay were shorter in the mini-laparotomy group (*p* = 0.02, *p* = 0.012, *p* = 0.006, respectively). The postoperative increments of IL-6(Δ2 h) in the mini-laparotomy group were significantly less than the increment in the non-mini-laparotomy group (*p* = 0.009). Clinical variables, including age, sex, cancer stage, cancer site, amount of blood loss, BMI, and methods of postoperative analgesia, showed no statistical differences between the two groups. Preoperative blood tests of CBC and biochemistry did not show significant differences between the two groups (data not shown).

**Table 7:** Effects of mini-laparotomy among patients receiving AR operation.

Variables	Wound length <7 cm	Wound length >7 cm	*p* values
Age (years)			
Median	56.50 (16)	63.50 (13)	0.132
Range	47–85	56–84	
Gender (M/F)	11/9	9/7	1.000
Cancer stage			0.624
Dukes’ A	1	2	
Dukes’ B	6	4	
Dukes’ C	13	13	
Dukes’ D	2	2	
Cancer sites			0.483
Sigmoid	5	6	
Rectum	15	10	
BMI (kg/m^2^)	23.52 (5)	23.30 (4)	0.14
Wound length (cm)	6.8 (0)	15.25 (9.6)	**<0.001**
Operation time (min)	150 (53)	215 (105)	**0**.**02**
Blood loss (ml)	200 (250)	225 (200)	0.912
Postoperative analgesia			0.196
PCA	15	15	
Non-PCA	5	1	
Ileus time (day)	2 (2)	4 (2)	**0**.**012**
Post-op stay (day)	9 (2)	13.38 (9)	**0**.**006**
IL-6 (pg/ml)			
Pre-op	0.00 (2.26)	0.98 (5.24)	0.369
Δ2 h	225.57 (258.91)	460.42 (665.23)	**0**.**009**
Δ24 h	102.67 (191.77)	117.47 (186.96)	0.671
Δ48 h	32.74 (44.46)	49.06(121.52)	0.386

Data of continuous variables were expressed as median (IQR). Mann-Whitney *U*-test for continuous data; Chi-square test for categorical data.

Significant differences in statistical comparisons are shown by bold values.

### 3.5 Effects of tumor characteristics and sites on serum IL-6 levels

We further investigated whether the characteristics of tumor and locations of tumor would influence serum levels of IL-6. We stratified patients based on stage of tumor, histological grade of cancer cells, or site of tumor, respectively, to compare the serum IL-6 levels between different patients groups. The pre-operative levels and the postoperative increments of serum IL-6 (Δ2 h, Δ24 h and Δ48 h) showed no significant differences between different patients groups stratified by stage of tumor, histologic grade, and site of tumor (Table 8).

**Table 8:** Effects of tumor characteristics on serum IL6 levels of patients (*n* = 43).

Variables	IL-6 pre-op median (IQR)	*p-*value*	IL-6(Δ2 h) median(IQR)	*p*-value*	IL-6(Δ24 h) median (IQR)	*p-*value*	IL-6(Δ48 h) median (IQR)	*p*-value*
Cancer stage^a^		0.426		0.671		0.098		0.612
Dukes’ A (*n* = 3)	0.00 (NA)		425.33 (NA)		102.67(NA)		62.97(NA)	
Dukes’ B (*n* = 10)	1.38 (7.83)		311.55 (381.36)		152.73 (178.68)		37.97 (55.66)	
Dukes’ C (*n* = 25)	0.00 (2.64)		236.22 (250.24)		48.95 (122.40)		42.55 (39.94)	
Dukes’ D (*n* = 5)	0.00 (26.19)		539.36 (727.21)		138.78 (621.34)		38.35 (376.21)	
Histological grade^b^		0.805		0.717		0.234		0.496
Well (*n* = 4)	2.14 (3.88)		231.87 (804.62)		194.95 (179.40)		34.00 (91.31)	
Moderately (*n* = 31)	0.00 (2.90)		293.68 (403.36)		101.81 (197.37)		39.88 (50.94)	
Poorly (*n* = 3)	1.74 (NA)		200.10 (NA)		36.33(NA)		14.73(NA)	
Missing data (*n* = 5)	0.00 (3.94)		181.23 (172.72)		150.96 (149.28)		53.92 (55.81)	
Tumor site^c^		0.783		0.290		0.760		0.900
Right colon (*n* = 6)	0.00 (3.35)		273.49 (268.94)		58.67 (164.40)		32.32 (46.39)	
Left colon (*n* = 12)	0.00 (4.58)		136.84 (323.23)		68.32 (304.43)		36.54 (168.92)	
Rectum (*n* = 25)	0.00 (3.28)		270.15 (340.49)		104.38 (182.12)		41.41(49.49)	

^a^ Dukes’ stage of colorectal cancer.

^b^ Histological grade: well differentiated, moderately differentiated, poorly differentiated, missing data (data of grade not mentioned in the pathological report).

^c^ Tumor site: anatomic location of colorectal cancer, right colon (cecum, ascending colon, hepatic flexure, and transverse colon), left colon (splenic flexure, descending colon, and sigmoid colon); IQR, interquartile range; NA, not available in SPSS.

* Kruskal Wallis test.

The pre-operative level and the postoperative increments of serum IL-6 showed no significant differences between different patients groups stratified by pathologic characteristics, including depth of tumor invasion, tumor size larger or smaller than median value, status of lymph node invasion, presence or absence of vascular invasion, presence or absence of lymphatic invasion, and presence or absence of perineurial invasion (data not shown).

## 4 Discussion

Our study is the first to demonstrate that abdominal wound length influences postoperative IL-6 levels and recovery of flatus passage after colorectal cancer resection. Notably, the IL-6 level at two hours after operation, in turn, influenced the recovery of flatus passage. These interactions influence the postoperative hospital stay. These findings are clinically relevant because faster recovery of bowel function and shorter postoperative hospital stays are important for enhanced recovery after surgery (ERAS).

The pathophysiology of POI after abdominal surgery has been proposed to be multifactorial and includes autonomic nerve activity, neurotransmitters, and bowel tissue inflammation. The type of anesthesia and analgesics, malnutrition, electrolyte imbalance, intra-abdominal infection, amount of blood loss, male sex, advanced age, respiratory insufficiency, and emergency surgery are risk factors for prolonged POI (1, 5). Recent studies have shown that mini-laparotomy (a smaller abdominal wound) and laparoscopic surgery are associated with earlier bowel motility (22–24). The influence of wound size on bowel motility is also supported by an animal study (25).

The ERAS protocol for colorectal surgery recommendations suggests the use of laparoscopic surgery for colorectal resection to achieve quicker recovery of bowel function and shorter hospital stay (26). In the Color II trial, laparoscopic surgery for rectal cancer, compared with conventional open surgery, yielded quicker recovery of bowel movement and shortened hospital stay (one-day reduction, respectively) (24). Hong et al. reported that laparoscopic resection of colorectal cancer shortened the time to first flatus passage by 1.2 days and hospital stay by four days compared to conventional open surgery (23). Our data and those of others have shown that mini-laparotomy surgery for colorectal cancer could shorten the time from surgery to the first flatus passage by 1–2 days and the postoperative stay by 3–5 days (18, 20). These data suggest that the mini-laparotomy and laparoscopic surgery have similar effects on earlier flatus passage and reduced postoperative hospital stay. This similar merit of mini-laparotomy and laparoscopic surgery can be explained by the smaller abdominal wounds compared with those of conventional open surgery.

Studies have reported that laparoscopic surgery for colorectal cancer, relative to open abdominal surgery, reduces the increase in serum IL-6 levels after surgery (27, 28). This reduction may be due to less trauma to the abdominal wall caused by laparoscopic surgery than by conventional open surgery (9, 11, 27, 28). Our study showed that mini-laparotomy surgery for colorectal cancer was significantly associated with decreased increment of IL-6 at 2 h after surgery [IL-6 (Δ2 h)]. The reduced increments in postoperative serum levels of IL-6, seen in both laparoscopic surgery and mini-laparotomy, can also be explained by smaller abdominal incision wounds. In our study, the decreased increment of IL-6, but not the decreased increment of IL-8, was significantly associated with earlier recovery from flatus passage and a shorter postoperative hospital stay. This finding is consistent with those of previous studies that reported an association between the inflammatory response to IL-6 and POI (9–12). This result also suggests that IL-6 influences not only the gastric emptying function but also colonic motility (13).

Using statistical analysis, our data showed a numeric correlation between wound length, recovery of the first flatus passage, and postoperative hospital stay. The results showed that one centimeter of wound length was associated with a waiting time of 0.151 days for the first flatus passage. In turn, a waiting time of one day for the first flatus passage was associated with a 1.012-day delay in the postoperative hospital stay. A similar study reported the effect of the length of the abdominal laparotomy wound on bowel function recovery. The authors estimated that an abdominal wound greater than 18 cm increased the waiting time of bowel movement by 0.5 days, compared to an abdominal wound length less than 18 cm. One centimeter increase in wound length correlated with a 2% increase in the delay of the first bowel movement (29). Our study further revealed a correlation between IL-6 increment and waiting time for the first flatus passage. A 1 pg/ml increment of IL-6(Δ2 h) was associated with 0.002 days of waiting time for the first flatus passage.

Studies have reported that surgery of the right colon has a higher chance of inducing delayed recovery of bowel function and prolonged ileus than surgery of the left-sided colon (30–32). One may wonder whether the different sites of mesenteric dissection or surgery in the small intestine influence clinical outcomes and IL-6 response. To exclude these potential confounding factors, we further analyzed the effects of mini-laparotomy in patients who underwent anterior resection in which the dissection of the mesentery was limited to the inferior mesenteric artery territory. The results suggested that the effects of mini-laparotomy remained the same with regard to a decrease in postoperative IL-6(Δ2 h) increment, a decrease in waiting time to have the first flatus passage, and a decrease in postoperative hospital stay. These results provide further evidence for the biological benefits of mini-laparotomy in the recovery of bowel function.

There have been studies reporting the alterations of serum IL-6 levels related to tumor characteristics of colorectal cancer. Concerning the association of IL-6 serum levels and tumor stage, one such study found a significant association of advanced staging of tumor and increasing IL-6 serum levels (33). On the contrary, another study did not find a significant association between tumor stage and serum levels of IL-6 (34). Tumor size and status of lymph node invasion have also been reported to be associated with serum levels of IL-6 (35). Our results did not show significant influences of these tumor characteristics on serum levels of IL-6. The discrepancy in results between our study and other studies may be related to 174 G>C polymorphism of IL-6 gene promoter (36). To further clarify this question, a prospective study with large numbers of patients to investigate the correlation between IL-6 promoter polymorphism and IL-6 serum levels is needed.

Surgical complications e.g., surgical site infection, anastomotic leakage, and sepsis, can occur after colorectal cancer resection (37, 38). These complications can cause local and systemic inflammation response, leading to elevated levels of IL-6 in the serum (39, 40). Recent studies showed that butyrylcholinesterase plasma level has emerged as a potential predictive marker for the surgical complications that occurred after colorectal cancer resection (41). Surgical complications are often associated with prolonged postoperative ileus and increased chance of morbidities (e.g., malnutrition, sepsis, and even mortality), leading to prolonged postoperative hospital stay and increased economic burden. Of note, the elevation of serum IL-6 elicited by surgical complications can cause misinterpretation of the IL-6 data in our study. To minimize the confounding effect caused by surgical complications, we have excluded patients who had surgical complications in this study. Thus the interpretation of our results should be applied only to patients who did not have surgical complications after the tumor resection surgery.

The strength of this study lies in the prospective collection of serum samples and clinical data. The high homogeneity of the patient sample, as the two surgeons who participated in this study, led to a consensus on the study design, surgical procedures, and postoperative care. Patients who developed complications or required colostomies were excluded from the final analysis. The limitations of this study are as follows: (1) the small sample size and patients enrolled only in a single hospital, (2) the physiological variations in cytokine levels between individuals, (3) the interpretation of our results only applied to patients who did not have surgical complications, and (4) lacking the information of 174 G>C polymorphism of IL-6 gene promoter. We have used the Δ values as described in the methodology in this study in the hope of minimizing the influence of physiological variations. Our study indicates the need for a prospective study enrolling large numbers of colorectal cancer patients who are willing to have IL-6 gene analysis and cytokine analysis during the treatment of tumor resection.

## 5 Conclusion

Our study provides novel findings to better understand the complex mechanisms of POI after surgical resection of colorectal cancer. These results suggest that smaller abdominal wounds and smaller postoperative IL-6 increments were associated with faster recovery from flatus passage and shorter hospital stays.

## Data Availability

The datasets presented in this article are not readily available because, under the institutional requirements, only the principal investigator is allowed to access the raw data of this study. Requests to access the datasets should be directed to the principal investigator M-JC via email: mjchen@mmh.org.tw.

## Supplementary material

Supplementary Figure S1 Median serum levels of IL-6, IL-8, and IL-10 are shown over time. Briefly, cytokines were quantified by ELISA in the serum collected preoperatively and at 2, 24, and 48 h after surgery.
