# Supplementary figures and images for: Abdominal wound length influences the postoperative serum level of interleukin-6 and recovery of flatus passage among patients with colorectal cancer
Source: Front Surg. 2024 Jun 24;11:1400264. doi: 10.3389/fsurg.2024.1400264 (PMC11228254; doi:10.3389/fsurg.2024.1400264)

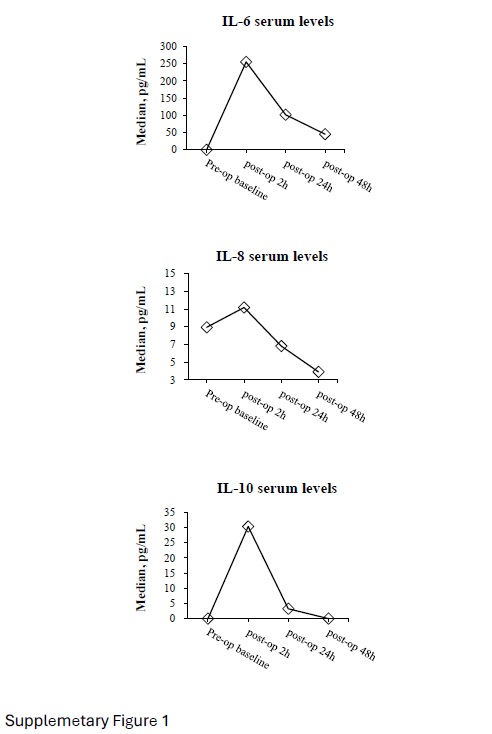

Supplement: Supplementary Figure S1 — Median serum levels of IL-6, IL-8, and IL-10 are shown over time. Briefly, cytokines were quantified by ELISA in the serum collected preoperatively and at 2, 24, and 48 h after surgery. [file Image1.jpg]
